# Enhancing betalains production and antioxidant activity in *Celosia argentea* cell suspension cultures using biotic and abiotic elicitors

**DOI:** 10.1038/s41598-024-83096-x

**Published:** 2025-01-02

**Authors:** Kanchanok Mueangnak, Haruthairat Kitwetcharoen, Sudarat Thanonkeo, Preekamol Klanrit, Jirawan Apiraksakorn, Poramaporn Klanrit, Poramate Klanrit, Pornthap Thanonkeo

**Affiliations:** 1 Department of Biotechnology, Faculty of Technology, Khon Kaen University, Khon Kaen, 40002 Thailand; 2 Walai Rukhavej Botanical Research Institute (WRBRI), Mahasarakham University, Maha Sarakham, 44150 Thailand; 3 Research Center for Value Added Agricultural Products (FerVAAPs), Khon Kaen University, Khon Kaen, 40002 Thailand; 4 Research Group of Chronic Inflammatory Oral Diseases and Systemic Diseases Associated with Oral Health, Department of Oral Biomedical Sciences, Faculty of Dentistry, Khon Kaen University, Khon Kaen, 40002 Thailand; 5 Department of System Biosciences and Computational Medicine, Faculty of Medicine, Khon Kaen University, Khon Kaen, 40002 Thailand

**Keywords:** Antioxidant activity, Betalains, Cell suspension culture, *Celosia argentea*, Elicitation, Plant secondary metabolites, Biotechnology, Plant sciences

## Abstract

*Celosia argentea* is a plant known for producing bioactive compounds, including betalains, which possess various biological and pharmaceutical properties. This study aimed to investigate the effect of biotic and abiotic elicitors on betalains production and their antioxidant activity in cell suspension cultures of *C. argentea*. Various concentrations of chitosan, yeast extract, salicylic acid, methyl jasmonate, copper sulfate (CuSO_4_), and cobalt chloride (CoCl_2_) were evaluated. The results revealed that chitosan, salicylic acid, methyl jasmonate, and CuSO_4_ significantly improved betalains production in the cell suspension cultures. Among these elicitors, chitosan at 5.0 mg/L and CuSO_4_ at 6.4 µM were the most effective in enhancing betalains production, yielding the highest concentrations of 4.65 and 4.99 mg/g dry weight, respectively. Notably, the betalains derived from the elicitor-treated cultures exhibited greater antioxidant activity compared to the control. These findings suggest that chitosan and CuSO_4_ are promising elicitors for sustainable in vitro production of betalains from *C. argentea* cell suspension cultures on a commercial scale, owing to their ability to enhance betalains production and antioxidant activity.

## Introduction

Betalains are nitrogen-containing, water-soluble, non-toxic major plant secondary metabolites derived from the metabolism of amino acid l-tyrosine. They are composed of two major groups: red-purple betacyanins and yellow-orange betaxanthins^1^. More than seventy betalain structures, including 42 betacyanins and 32 betaxanthins, have been characterized^2^. Compared to anthocyanins, betalains are more water-soluble, have greater dyeing capacity, and are more stable in a pH range of 3 to 7 ^2,3^, making them potentially useful in diverse applications such as natural colorants in foods and beverages, dyeing agents in textiles and cosmetics, and film materials for coating metal surfaces. Furthermore, betalains exhibit several biological properties, including anti-inflammatory, antioxidant, anticancer, and antimicrobial activities, indicating their potential for pharmaceutical and medical applications^1^.

Plants belonging to the families Amaranthaceae, Basellaceae, Cactaceae, Portulacaceae, and Nyctaginaceae are the main sources of betalains^1^. Recently, red beetroot (*Beta vulgaris*) has become the major commercial source for betalains production^4^. However, there are limitations to using this crop, such as the limited color range of the pigment, the presence of earthy-musty odorants (geosmin and pyrazines), and a high content of nitrates, which are precursors of carcinogenic nitrosamines^5^. Therefore, the search for alternative potential betalains sources that can offer safer compounds with a greater pigment color range and wider sources for sustainable betalains production is of great importance.

*Celosia argentea*, also known as cockscomb, is an annual dicotyledonous ornamental plant belonging to the Amaranthaceae family. This plant is native to Africa, South America, and Southeast Asia; it is widely distributed throughout subtropical and temperate zones. Apart from being used as a vegetable, *C. argentea* has also been widely utilized in traditional medicine to treat various disorders, such as fever, diarrhea, piles, itching, wounds, jaundice, gonorrhea, and rheumatoid arthritis. This plant produces and accumulates a variety of bioactive compounds, including phenolics, tannins, saponins, flavonoids, alkaloids, steroids, and betalains, conferring several biological and pharmaceutical properties, such as anti-inflammatory, anticancer, antimicrobial, antioxidant, antidiabetic, and other health benefits like immunological activity, cytoprotective, and hepatoprotective effects^6,7^. Previous research by Schliemann et al.^8^ revealed that *C. argentea* contained 0.157 g/g fresh weight (FW) of total betalains in yellow inflorescence and 0.293 g/g FW of total betalains in orange-red inflorescence, making it a promising alternative source for commercial betalains production.

However, the production of betalains from field-cultivated *C. argentea* plants is laborious, requires large areas, and is highly dependent on seasonal, climatic, and geographical conditions. Additionally, infection with plant pathogens can also lower the quality and yield of betalains. Plant cell cultures offer a promising biotechnological platform for the production of plant secondary metabolites, overcoming the limitations of field-cultivated plants and providing a stable, economical, and sustainable supply of products. Several studies have reported the production of betalains by plant cell cultures in different plant species, such as *B. vulgaris*, *Hylocereus costaricensis*, *Bougainvillea* spp., *Pereskia aculeata*, and *C. cristata*^9−15^. However, the productivity and yield of betalains are still low as these bioactive compounds are not essential for plant growth and development compared to primary metabolites.

Several strategies have been applied to enhance the production of plant secondary metabolites, and elicitation, either biotic or abiotic, represents the most effective and promising technique for increasing phytoconstituent production, including betalains. A diverse range of biotic elicitors, such as chitosan, yeast extract, β-glucan, and microbial polysaccharides, and abiotic elicitors, such as microelements, light, and plant phytohormones, have been successfully used to improve the production of many plant secondary metabolites^14−25^. However, there is a lack of information on the biological effect of biotic and abiotic elicitation on betalains production in cell suspension cultures of *C. argentea.*

Therefore, the present work aims to evaluate the effect of some elicitors, including chitosan, yeast extract, methyl jasmonate, salicylic acid, copper sulfate (CuSO_4_), and cobalt chloride (CoCl_2_), on the in vitro production of betalains from *C. argentea* using a cell suspension culture system as a model. The results obtained in this study, which have never been recorded before, will contribute to the development of a reliable and effective protocol for the sustainable production of betalains on a commercial scale.

## Materials and methods

### Chemicals

Murashige and Skoog (MS) basal medium, solidifying agents, and plant growth regulators (PGRs), including 6-benzylaminopurine (BAP) and 2,4-dichlorophenoxyacetic acid (2,4-D), were purchased from PhytoTech Labs, Lenexa, KS, USA. Elicitors, such as chitosan, salicylic acid, and methyl jasmonate, were obtained from Sigma Aldrich Corporation, Burlington, MA, USA. Yeast extract was acquired from Titan Biotech Ltd., Bhiwandi, Rajasthan, India. Other chemicals, including copper sulfate (CuSO_4_), cobalt chloride (CoCl_2_), hydrochloric acid (HCl), sodium hydroxide (NaOH), and sucrose, were purchased from a local supplier in Khon Kaen province, Thailand.

## Plant material

The friable callus of *C. argentea* used in this study was kindly provided by Dr. Preekamol Klanrit, Khon Kaen University, Thailand. The callus was maintained on MS basal medium containing 30 g/L sucrose and supplemented with 0.1 mg/L BAP and 1 mg/L 2,4-D. The callus culture was incubated in a standard cultivation room at 25 ± 2 °C with 3,000 lx light intensity, 16 h light/8 h dark photoperiod, and 80% relative humidity. The callus was subcultured onto fresh MS basal medium every 14 days.

## Cell suspension cultures

Two grams of fresh weight of 14-day-old callus cultures of *C. argentea* were transferred into 250-mL Erlenmeyer flasks containing 100 mL liquid MS basal medium with 30 g/L sucrose, 0.1 mg/L BAP, and 1 mg/L 2,4-D. All suspension cultures were incubated on a rotary incubator shaker at 120 rpm in a standard cultivation room at 25 ± 2 °C with 3000 lx light intensity, 16 h light/8 h dark photoperiod, and 80% relative humidity. The growth and total betalains production of the *C. argentea* suspension cultures were monitored during cultivation.

## Effect of biotic elicitors on total betalains production

Biotic elicitors used in this study included chitosan and yeast extract. Stock solutions of chitosan and yeast extract were prepared using the protocol described by Shah et al.^21^. Chitosan was dissolved in 0.1% (v/v) acetic acid, while yeast extract was dissolved in sterile distilled water. After complete dissolution, they were filter-sterilized through a 0.45 μm filter membrane (Minisart^®^ NML, Sartorius Stedim Biotech GmbH, Goettingen, Germany). The concentrations of chitosan tested were 1.0, 2.5, 5.0, 10.0, and 20.0 mg/L^21^, while those of yeast extract were 100, 200, 300, and 400 mg/L^14^. The same volume (1 mL of each elicitor solution) was separately introduced into the suspension cultures of *C. argentea* at the exponential growth phase (on day 8 after cultivation), and the treated suspension cultures were subsequently incubated on a rotary incubator shaker at 120 rpm in a standard cultivation room at 25 ± 2 °C with 3000 lx light intensity, 16 h light/8 h dark photoperiod, and 80% relative humidity. Based on preliminary findings, betalain production increased rapidly after elicitor addition, peaking at day 4 of cultivation. Extended incubation periods led to decreased cell growth and betalain yields. Thus, this study collected biotic elicitor-treated suspension cells on day 4 post-elicitation to determine total betalain production. The suspension cultures without adding chitosan and yeast extract (addition of 1 mL sterile distilled water) served as controls.

## Effect of abiotic elicitors on total betalains production

Abiotic elicitors tested in the current study included salicylic acid, methyl jasmonate, CuSO_4_, and CoCl_2_. Salicylic acid was prepared by dissolving in 1% (v/v) ethanol using the protocol described by Mendoza et al.^22^, while methyl jasmonate, CuSO_4_, and CoCl_2_ were prepared by dissolving in sterile distilled water. All stock solutions of abiotic elicitors were filter-sterilized through a 0.45 μm filter membrane (Minisart^®^ NML, Sartorius Stedim Biotech GmbH, Goettingen, Germany). The different concentrations of abiotic elicitors tested were: 25, 50, 100, and 200 µM for salicylic acid; 50, 100, 150, and 200 µM for methyl jasmonate; 1.6, 3.2, 6.4, and 12.8 µM for CuSO_4_; 1.68, 3.35, 6.70, and 13.40 µM for CoCl_2_. The same volume (1 mL of each elicitor solution) was separately introduced into the suspension cultures of *C. argentea* at the exponential growth phase (on day 8 after cultivation), and the treated suspension cultures were subsequently incubated under the same conditions as mentioned in the biotic elicitor experiment. After 4 days of cultivation, the abiotic elicitor-treated suspension cells were collected, and total betalains production was determined. The suspension cultures without adding salicylic acid, methyl jasmonate, CuSO_4_, and CoCl_2_ (addition of 1 mL sterile distilled water) served as controls.

### Betalains extraction and quantification

After filtration using filter paper (Whatman No. 1, Cytiva, China), the collected suspension cells were dried in a hot air oven (Model FD240, WTB Binder, Tuttlingen, Germany) at 45 °C until constant weight. The resulting dried cells were ground using a mortar and pestle, and the resulting powders were subjected to betalains extraction using the protocol described by Henarejos-Esudero et al.^26^, Winson et al.^15^, and Visockis et al.^27^ with some modifications. Briefly, 2 g of dried powder was soaked in 100 mL of deionized water (pH 5.8) and incubated in a controlled incubator shaker (JSR, Gongju, Republic of Korea) at 50 °C and 150 rpm. After 1 h of incubation, the supernatants were collected by filtration and centrifugation at 5,000 rpm for 5 min at room temperature. The concentrations of betacyanin, betaxanthin, and total betalains in the resulting supernatants were determined using a spectrophotometric method described by Visockis et al.^27^. The betacyanin absorption was measured at 535 nm, while that of betaxanthin was at 483 nm using a spectrophotometer (Libra S6+, Biochrom, Harvard Bioscience, Inc., Cambridge, UK). The total betalains content was calculated as the sum of betacyanin (BC) and betaxanthin (BX) according to the Eqs. (1) and (2) and was expressed as mg/g dry weight (DW).

$$\:BC\:or\:BX\:\left(\frac{\text{m}\text{g}}{\text{g}}\right)=\frac{A\:\times\:DF\:\times\:MW\:\times\:V\:\times\:\text{1,000}}{\\\epsilon\:\times\:L\:\times\:m}\dots\:\dots\:\dots\:\dots\:\dots\:\dots\:\left(1\right)$$

$$\:Total\:betalain\:content\:\left(\frac{\text{m}\text{g}}{\text{g}}\right)=BC+BX\dots\:\dots\:\dots\:\dots\:\dots\:\dots\:\left(2\right)$$

Where *A* is the absorption value of betacyanin and betaxanthin at the wavelengths of 535 and 483 nm, respectively; *DF* is the dilution factor; *MW* is the molecular weight of betacyanin (550 g/mol) and betaxanthin (308 g/mol); *V* is the volume of sample solution (L); *ε* is the extinction coefficient of betacyanin (60,000 L/mol.cm in water) and betaxanthin (48,000 L/mol.cm in water); *L* is the path length of the cuvette (1 cm), and *m* is the weight of dried sample (g).

Betalains were also extracted from 3-month-old flowers of field-cultivated *C. argentea*. After harvesting, the flowers were dried in a hot air oven (Model FD240, WTB Binder, Tuttlingen, Germany) at 45 °C until constant weight and ground using a mortar and pestle. The resulting powders were subjected to betalains extraction and quantification using the same procedures mentioned earlier.

### Antioxidant activity of betalains from ***C. argentea***.

The antioxidant activity of betalains extracted from suspension cultures of *C. argentea* treated with different biotic and abiotic elicitors was determined using the 2,2−diphenyl−1−picrylhydrazyl (DPPH) and 2,2’−azino−bis (3−ethylbenzothiazoline−6−sulfonic acid) (ABTS) assays, following the protocol described by Shah et al.^21^. For extraction, 2 g of dried powder of *C. argentea* suspension cells was incubated in 100 mL of deionized water (pH 5.8) at 50 °C and 150 rpm for 1 h. After filtration and centrifugation at 5,000 rpm for 5 min at room temperature, the crude extract was subjected to antioxidant activity analysis. For the DPPH assay, 75 µL of sample extract (diluted 10 times) was mixed with 225 µL of 0.1 mM DPPH solution and then incubated in the dark at 25 ± 2 °C for 30 min. For the ABTS assay, 10 µL of undiluted sample extract was mixed with 190 µL of ABTS solution and then incubated in the dark at 25 ± 2 °C for 6 min. A Tecan Infinite 200 Pro microplate reader (Tecan Austria GmbH, Grödig, Austria) was used to monitor the absorbance at 517 nm for the DPPH assay and 734 nm for the ABTS assay. The DPPH assay results were expressed as a percentage of free radical scavenging activity, which was calculated using the following equation:

$$\:DPPH\:free\:radical\:scavenging\:activity\:\left(\text{\%}\right)=\frac{A\ control\:-\:A\:sample}{A\:control}\:\times100$$

For the ABTS assay, the data was expressed as a percentage of ABTS^+^ inhibition, which was calculated using the following equation:

$$\:{ABTS}^{+}inhibition\:\left(\%\right)=\frac{A\:control\:-\:A\:sample}{A\:control}\:\times100$$

Where *A*_*control*_ is the absorbance of the reaction mixture without the sample solution, and *A*_*sample*_ is the absorbance of the reaction mixture with the sample solution.

## Experimental design and data analysis

All experiments were carried out at least twice, each with five replications, using a statistical model based on a completely randomized design (CRD). The experimental data were statistically analyzed using IBM SPSS Statistics 28 for Windows (IBM Corporation, Armonk, NY, USA). Differences between treatments were evaluated using Duncan’s multiple-range test (DMRT), with statistical significance set at *p* ≤ 0.05. Results are presented as means ± standard deviations (SDs).

## Results and discussion

### Growth profile and biomass production of *C. argentea *cell suspension cultures.

Plant secondary metabolites can be produced in vitro using a plant cell callus or cell suspension culture system under controlled and aseptic cultivation conditions. However, due to several advantages such as rapid growth rate, high biomass yield, uniform cells with high genetic stability, easy harvesting, and ease of scale-up for biomass production, a cell suspension culture system is widely used for commercial purposes^21,28^.

**Fig. 1:**
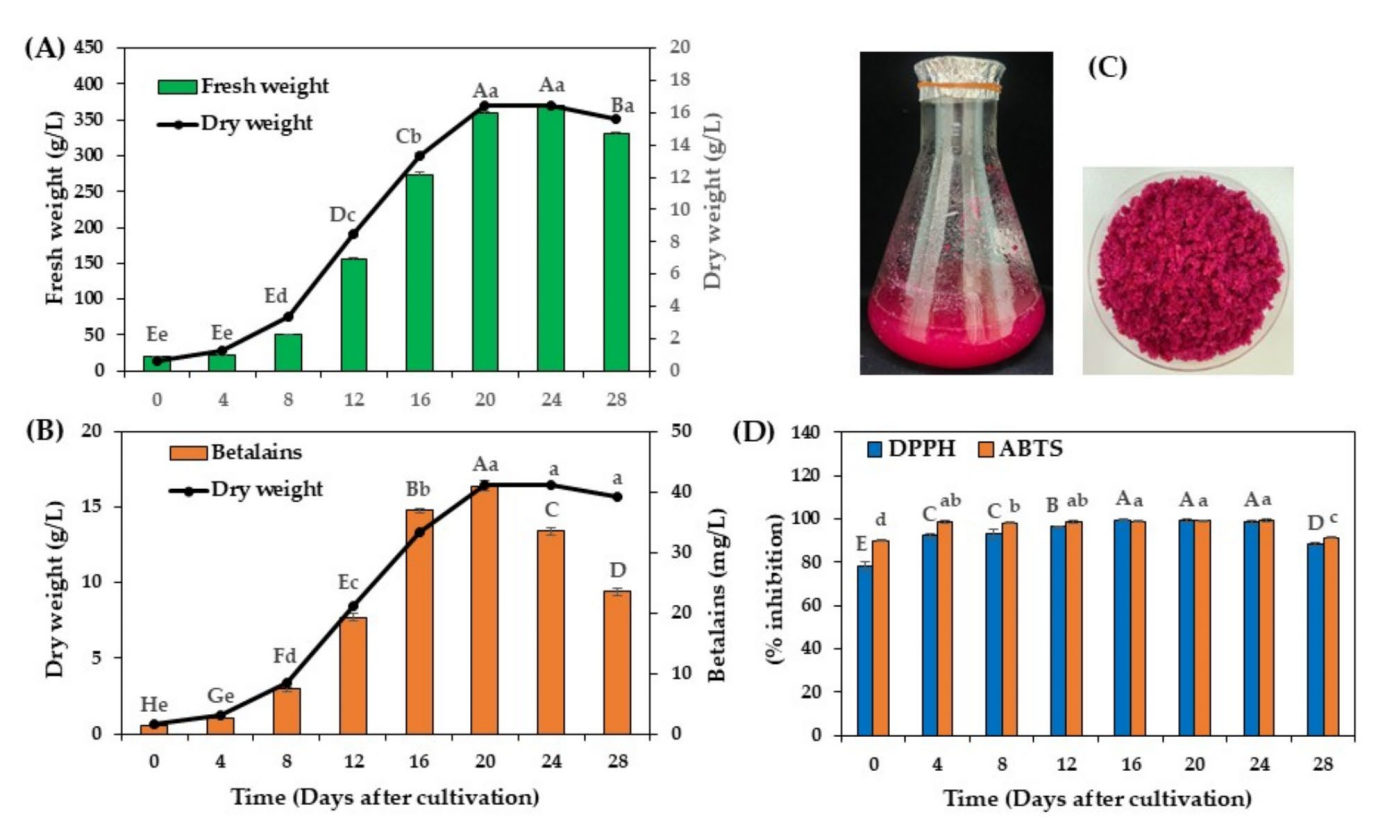
Growth profile and total betalains production of cell suspension cultures of *C. argentea* in liquid MS basal medium with 30 g/L sucrose, 0.1 mg/L BAP, and 1 mg/L 2,4-D. (**A**) Fresh and dry weight of cell suspension cultures, (B) Dry weight and total betalains production, (C) suspended cells of *C. argentea* after cultivation for 24 days, and (D) antioxidant activity of betalains extracted from cell suspension cultures of *C. argentea*. Bars and lines represent means ± standard deviations (SDs) from two independent experiments (*n* = 5). Figure 1A, bars (fresh weight) following different uppercase letters and lines (dry weight) following different lowercase letters indicate statistically significant differences between samples. Figure 1B, bars (betalains) following different uppercase letters and lines (dry weight) following different lowercase letters indicate statistically significant differences between samples. Figure 1D, for each parameter, different letters above the bars indicate statistically significant differences between samples. All statistical analyses were performed using Duncan’s Multiple Range Test (DMRT) at *p* ≤ 0.05.

The growth profile, biomass accumulation, and betalains production of *C. argentea* cell suspension cultures in liquid MS basal medium containing 30 g/L sucrose, 0.1 mg/L BAP, and 1 mg/L 2,4-D were assessed. As shown in Fig. 1A, a slight increase in biomass was observed during the first 4 days, an adaptation stage of cells to new environmental conditions. After 4 days, a dramatic increase in cells was detected, with the maximum fresh weight (FW) and dry weight (DW) reaching approximately 370 and 16.45 g/L, respectively, on day 24. A slight decrease in FW and DW was observed after that, possibly due to nutrient depletion.

The production and accumulation of total betalains in the cell suspension cultures were closely correlated with the growth profile (Fig. 1B). This finding suggested that the production of betalains in *C. argentea* cell suspension cultures is growth-associated, similar to other plant secondary metabolites previously reported, such as berberine in *Thalictrum regosum*^29^, anthocyanins in *Angelica archangelica*^30^, and alkaloids in *Catharanthus roseus* cell suspension cultures^31^. The production of total betalains reached its peak on day 20, with a maximum concentration of 40.93 mg/L (equivalent to 2.49 mg/g dry weight) and a productivity of 0.13 mg/g per day. However, after this point, both the production and accumulation of total betalains declined, coinciding with a decrease in plant cell growth. This reduction may be attributed to the release of betalains from dead and damaged cells. Evidence of this phenomenon was observed on day 24, as shown in Fig. 1C, where the culture medium displayed a distinctive red coloration due to the presence of betalains from lysed cells.

Betalains are known to possess several biological properties, including antioxidant activity. They can directly scavenge free radicals and indirectly activate antioxidant defense mechanisms, such as antioxidant enzymes or extracellular signal-regulated kinase proteins^1^. In this study, the antioxidant activity of betalains extracted from cell suspension cultures of *C. argentea* was determined using DPPH and ABTS assays (Fig. 1D). A close correlation between antioxidant activity and total betalains content was observed, with the maximum antioxidant activities being approximately 99.63% radical scavenging activity and 99.28% inhibition for the DPPH and ABTS assays, respectively.

Analysis of betalains from 3-month-old field-cultivated *C. argentea* flowers revealed a maximum concentration of 2.95 mg/g DW and productivity of 0.03 mg/g.day. Despite the floral betalain content being 1.2-fold higher, its productivity was 4.3-fold lower than cell suspension cultures. Floral betalains exhibited slightly reduced antioxidant activities (91.26% DPPH scavenging, 89.29% ABTS inhibition) compared to suspension culture-derived betalains. These findings highlight the potential of *C. argentea* cell suspension cultures as an efficient, sustainable platform for producing betalains with enhanced antioxidant properties.

### Effect of biotic elicitors on total betalains production in cell suspension cultures of *C.argentea*.

During plant growth and development, the production and accumulation of secondary metabolites are relatively low, as most of these compounds are not essential for growth and development. Elicitation using various types of elicitors is recognized as one of the most effective techniques for improving the biotechnological production of plant secondary metabolites^17^. Elicitors can be classified as biotic or abiotic, and their effects vary among plant species, growth stages, and cultivation conditions. In this study, the biotic elicitors chitosan and yeast extract were assessed for their effect on betalains production in cell suspension cultures of *C. argentea*.

Preliminary studies showed that applying elicitors at the start of cultivation (day 0) resulted in lower biomass accumulation and betalains production compared to application at the beginning of the exponential growth phase (day 8). This is likely because precursors for secondary metabolite synthesis, particularly primary metabolites, are limited during the initial growth phase as they are utilized for biomass formation ^17,32,33^. Therefore, in the current study, elicitors were applied to the culture medium on day 8, when biomass and betalains production were noticeably increased (Fig. 1).

Chitosan, a linear D-(1,4)-glucosamine polymer and cationic polysaccharide, is a structural component of animal and plant fungal pathogens. It affects various plant physiological processes, including growth, development, and morphogenesis ^34^. It has been widely used as a biotic elicitor to enhance the in vitro production of various plant secondary metabolites, such as plumbagin^35^, stilbenes^36^, silymarin^37^, and flavonolignans ^21^, and the production of these bioactive compounds appears to be dependent on the concentration of chitosan tested. Here, the effect of chitosan on betalains production in *C. argentea* suspension cultures was tested using different concentrations based on the study by Shah et al.^21^.

**Fig. 2:**
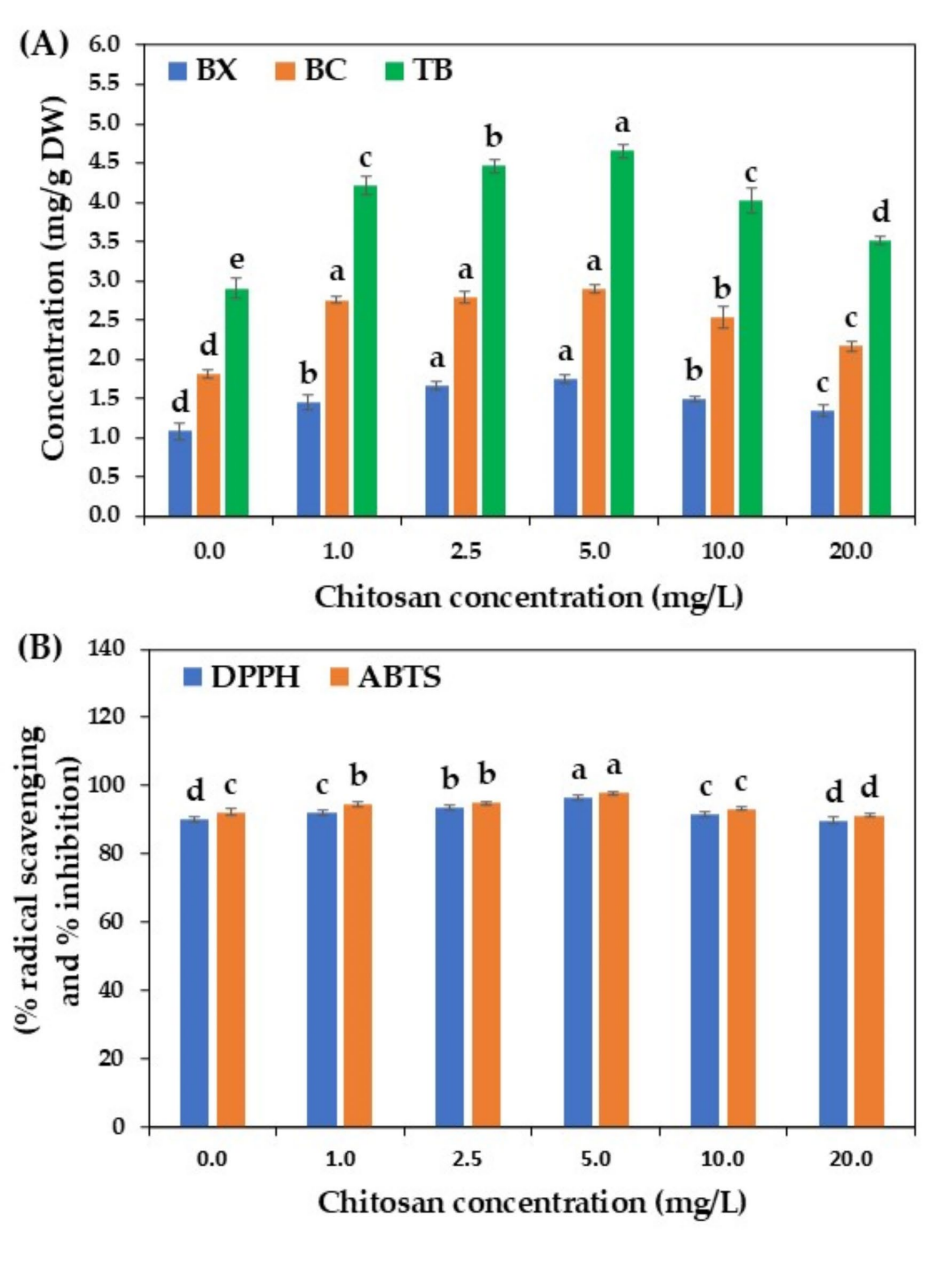
Effect of chitosan on betalains production by cell suspension cultures of *C. argentea*. (A) betalains production and (B) antioxidant activity. BX is betaxanthin, BC is betacyanin, and TB is total betalains. Bars represent means ± standard deviations (SDs) from two independent experiments, each with five replications. For each parameter, different letters above the bars indicate statistically significant differences between treatments (*p* < 0.05).

As shown in Fig. 2A, low concentrations of chitosan promoted betalains production and accumulation. The highest total betalains content of 4.65 mg/g DW was detected in 5.0 mg/L chitosan-elicited cultured cells, approximately 1.6-fold higher than the control. Chitosan is involved in plant defense responses by inducing the synthesis of pathogenesis-related proteins, defense enzymes (e.g., phenylalanine ammonia-lyase, catalase, peroxidase), and secondary metabolites^17,18,21,34,36^. The enhanced betalains production in *C. argentea* suspension cultures may be associated with the defense response mechanism triggered by chitosan elicitation. Notably, high concentrations of chitosan tended to reduce betalains production, suggesting a dose-dependent effect. Previous studies also reported that high chitosan concentrations can negatively impact enzyme activities involved in defense responses and secondary metabolite biosynthesis pathways, resulting in reduced metabolite production, such as phenolic compounds and rosmarinic acid ^18,38^.

The betalains produced from chitosan-treated *C. argentea* suspension cells exhibited high antioxidant activity, correlated with their content (Fig. 2B). The highest DPPH and ABTS antioxidant activities (96.47% radical scavenging and 97.82% inhibition, respectively) were observed with 5.0 mg/L chitosan treatment, followed by 2.5 mg/L. These findings suggest that chitosan improved betalains production and accumulation, consequently enhancing their antioxidant activity, similar to the study by Shah et al.^21^.

**Fig. 3:**
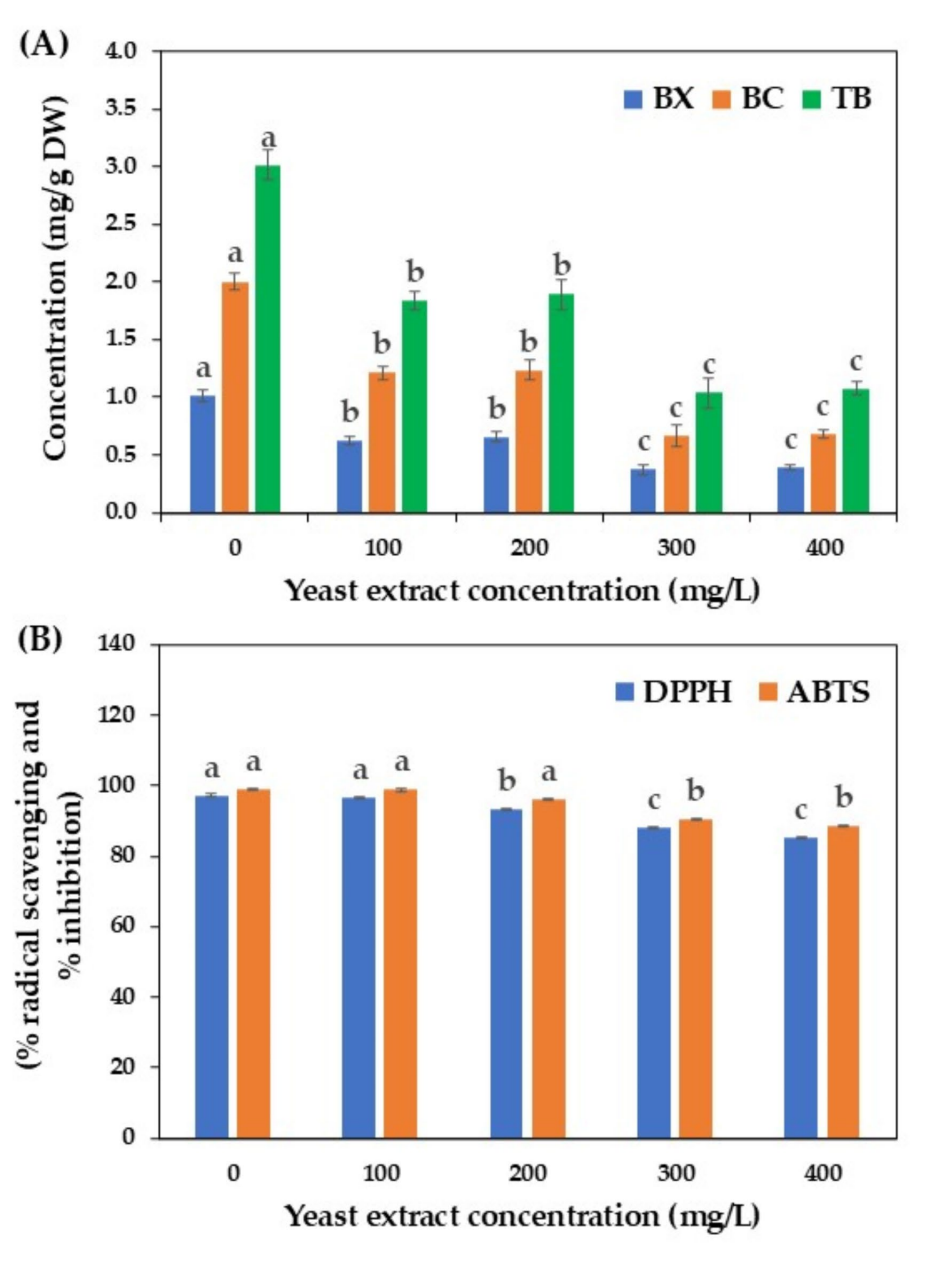
Effect of yeast extract on betalains production by cell suspension cultures of *C. argentea*. (A) betalains production and (B) antioxidant activity. BX is betaxanthin, BC is betacyanin, and TB is total betalains. Bars represent means ± standard deviations (SDs) from two independent experiments, each with five replications. For each parameter, different letters above the bars indicate statistically significant differences between treatments (*p* < 0.05).

Yeast extract, a rich source of nucleotides, proteins, amino acids, sugars, and trace elements^39^, has been used as a biotic elicitor to enhance the in vitro production of various plant secondary metabolites, including podophyllotoxin in *Linum album*^40^, silymarin in *Silybum marianum*^41^, stilbenes in *Cayratia trifolia*^42^, and betalains in *C. cristata* and *H. costaricensis*^14,15^. However, no information was available on the effect of yeast extract on betalains production in *C. argentea* cell suspension cultures. Therefore, various concentrations of yeast extract (100, 200, 300, and 400 mg/L), based on the study by Warhade and Badere ^14^, were tested (Fig. 3A).

Contrary to expectations, the addition of yeast extract did not exert a positive impact on betalains production in *C. argentea* suspension cultures. As yeast extract concentrations increased, the contents of betaxanthin, betacyanin, and total betalains decreased compared to the control treatment without yeast extract supplementation. Similar results were reported for betalains production in *C. cristata* cell suspension cultures^14^ and total phenolic and flavonoid production in *Phoenix dactylifera* cell suspension cultures^19^ after yeast extract elicitation. While yeast extract is known to trigger defense response mechanisms and enhance secondary metabolite production in some plant species, high concentrations may negatively impact enzyme activities involved in defense responses, secondary metabolite biosynthesis pathways, and overall betalains production.

Notably, these results contrast with those of Winson et al.^15^, who demonstrated that yeast extract concentrations between 50 and 200 mg/L promoted betalains production and accumulation in *H. costaricensis* callus cultures. These findings suggest that different plant species and culture systems respond differently to yeast extract elicitation.

Figure 3B summarizes the antioxidant properties of betalains isolated from *C. argentea* cell suspension cultures after yeast extract elicitation. Relatively high DPPH and ABTS antioxidant activities were observed in all treatments, with the highest activities detected in the control treatment without yeast extract addition. A slight decrease in antioxidant activity was observed with increasing yeast extract concentrations, correlated with the reduction in betalains production. These findings are consistent with those reported by Al-Khayri and Naik^19^ and Winson et al.^15^.

### Effect of abiotic elicitors on total betalains production from cell suspension cultures of *C. argentea*.

In addition to biotic elicitation, the effects of abiotic elicitors, including salicylic acid, methyl jasmonate, CuSO₄, and CoCl₂, were also investigated for their impact on betalains production in cell suspension cultures of *C. argentea*. A literature review revealed that salicylic acid and methyl jasmonate, also known as phytohormones, are classified as abiotic elicitors^24^.

Salicylic acid, a phenolic compound, plays a crucial role in plant defense regulatory systems. It is involved in various developmental and physiological processes, such as plant growth and development, stomatal movements, photosynthesis, ethylene production, enzyme activity, and pigment accumulation^43^. The accumulation of salicylic acid upon infection by plant pathogens or stressful environmental conditions, like cold, drought, and salinity, can trigger a wide range of defense responses, including the production of various plant secondary metabolites^17,24,44^. Salicylic acid has been widely used as an elicitor to enhance the production of several bioactive compounds, such as stilbene in *Vitis vinifera*^45^, camphor and phenolic compounds in *Achillea gypsicola*^46^, bacoside A in *Bacopa monnieri*^47^, phenolic compounds in *P. dactylifera*^19^, and rhamnetin in *Vernonia anthelmintica* cell suspension cultures^48^.

**Fig. 4:**
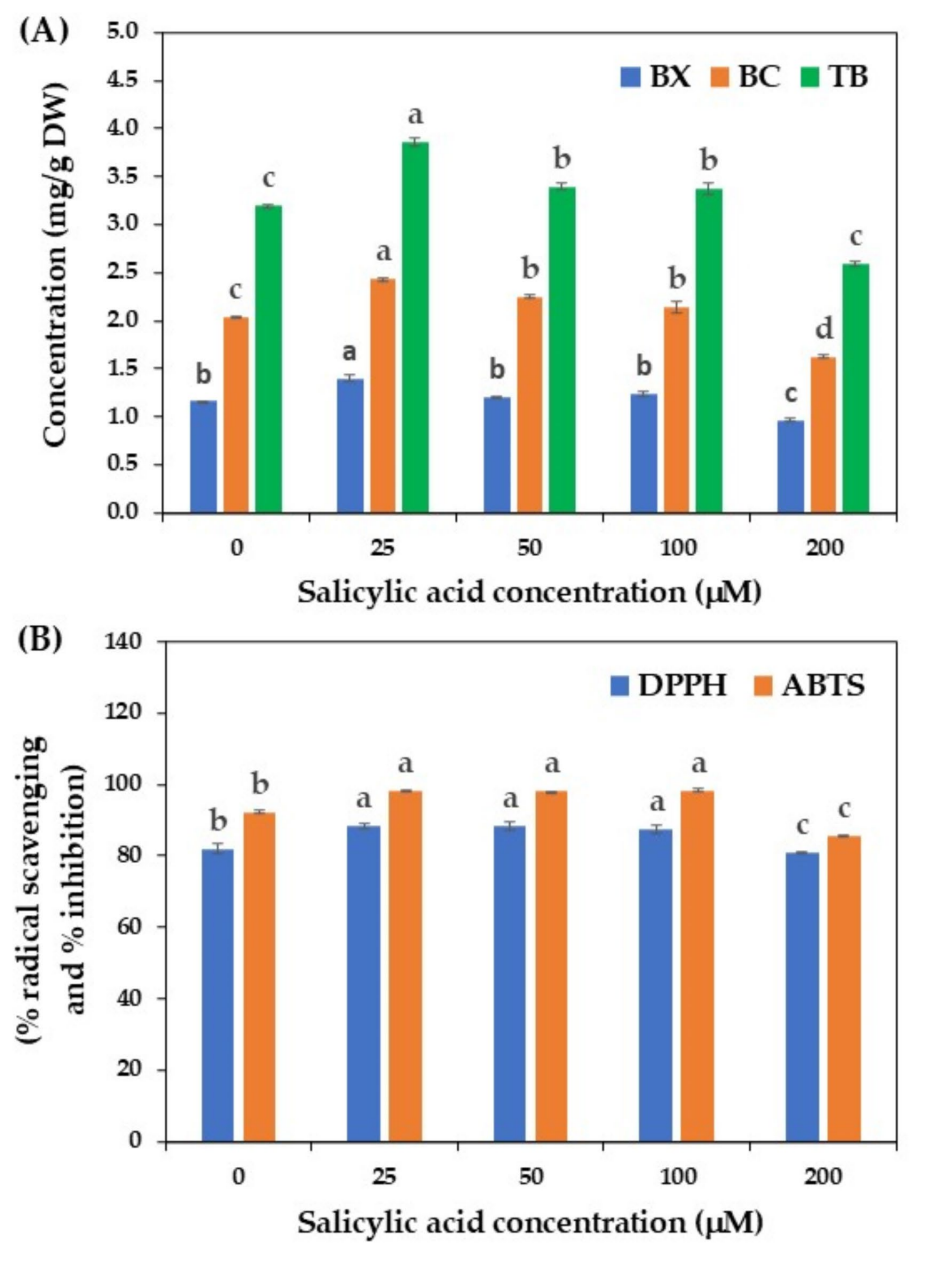
Effect of salicylic acid on betalains production by cell suspension cultures of *C. argentea*. (**A**) betalains production and (**B**) antioxidant activity. BX is betaxanthin, BC is betacyanin, and TB is total betalains. Bars represent means ± standard deviations (SDs) from two independent experiments, each with five replications. For each parameter, different letters above the bars indicate statistically significant differences between treatments (*p* < 0.05).

Based on the literature review, no information was available on the application of salicylic acid to enhance betalains production in cell suspension cultures of *C. argentea*. Therefore, the effect of salicylic acid at various concentrations, based on a study by Al-Khayri and Naik^19^, on betalains production was tested in this study. A significant increase in betalains production was observed in cell cultures treated with 25, 50, and 100 µM salicylic acid (Fig. 4A). The highest content of total betalains (3.86 mg/g DW) was found when cells were treated with 25 µM salicylic acid, approximately 1.2-fold higher than the control cells without salicylic acid treatment. Increasing the concentrations of salicylic acid from 100 µM to 200 µM resulted in a reduction of betalains production. These findings align with a study by Al-Khayri and Naik^19^, who reported that salicylic acid at a low concentration (50 mg/L) promotes the production of phenolic compounds in cell suspension cultures of *P. dactylifera*, whereas high concentrations of salicylic acid reduced the phenolic content. In red beetroot, the direct application of salicylic acid at 1 mmol/L also enhanced the betaxanthin, betacyanin, and total betalains content in potted plants, and the content of these compounds reduced upon increasing the concentrations of salicylic acid. One possible explanation is that high concentrations of salicylic acid may interfere with the expression of genes or the action of enzymes involved in the phenylpropanoid and shikimic acid pathways, which are responsible for the production of several secondary metabolites, including betalains^25,49^.

Figure 4B presents the antioxidant activities of betalains extracted from salicylic acid-treated cell cultures of *C. argentea* based on DPPH and ABTS assays. A close correlation was observed between betalains content and antioxidant activity, with high DPPH and ABTS activities detected in betalains samples extracted from treated cells with salicylic acid in the range of 25 to 100 µM. A relatively low level of antioxidant activity was found in control cells and cells treated with a high concentration of salicylic acid (200 µM). A similar result was reported in cell suspension cultures of *P. dactylifera* after salicylic acid elicitation at high levels^19^.

Notably, the observed difference between DPPH and ABTS assay results of salicylic acid treatment may be attributed to the role of salicylic acid as a plant signaling molecule. Salicylic acid can trigger various defense mechanisms, including the production of phenolic compounds, flavonoids, and antioxidant enzymes (e.g., superoxide dismutase, catalase, peroxidases), in addition to betalains^43^. These salicylic acid-induced antioxidants often demonstrate higher reactivity with ABTS compared to DPPH. This differential reactivity could explain the higher activity values observed in the ABTS assay.

Methyl jasmonate, a volatile methyl ester belonging to the jasmonate family, is a significant phytohormone that plays an important role in various plant physiologies. It can act as a signaling molecule in response to biotic and abiotic stresses. Due to its volatile nature, methyl jasmonate can facilitate intra- and intercellular communication in plants, regulating defense responses and consequently enhancing the production of several plant secondary metabolites^24,50,51^.

**Fig. 5:**
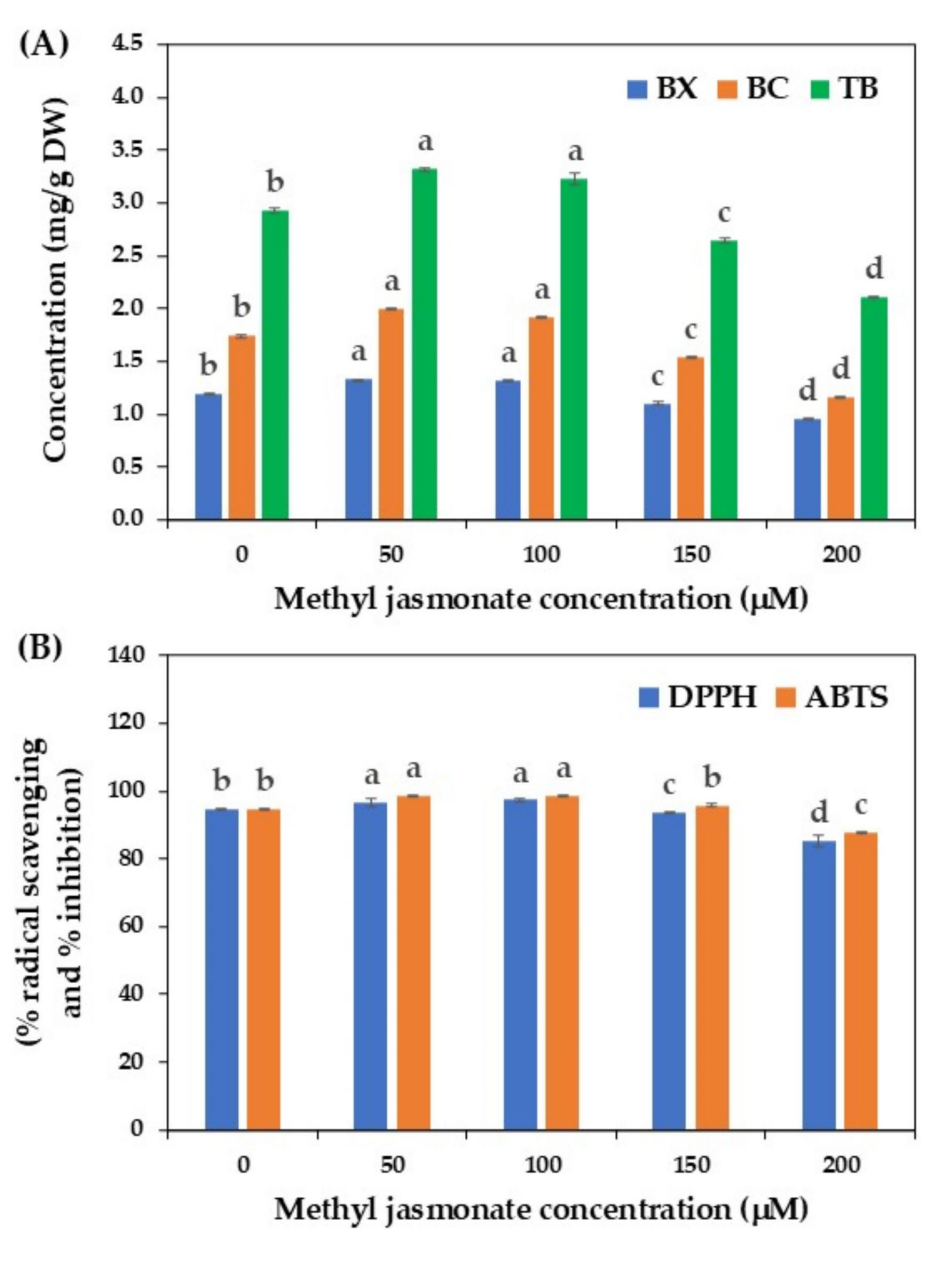
Effect of methyl jasmonate on betalains production by cell suspension cultures of *C. argentea*. (A) betalains production and (B) antioxidant activity. BX is betaxanthin, BC is betacyanin, and TB is total betalains. Bars represent means ± standard deviations (SDs) from two independent experiments, each with five replications. For each parameter, different letters above the bars indicate statistically significant differences between treatments (*p* < 0.05).

Methyl jasmonate has been successfully utilized to improve the production of various bioactive compounds in different plant species, such as gymnemic acid in *Gymnema sylvestre*^52^, hyperin and quercetin in *Hypericum perforatum*^53^, camphor and phenolic compounds in *A. gypsicola*^46^, rhamnetin in *V. anthelmintica* ^48^, and eugenol, phenolic and flavonoid in *Ocimum sanctum* cell cultures^23^. However, no reports have been conducted on the effect of methyl jasmonate in cell suspension cultures of *C. argentea*. Therefore, this study assessed the production of betalains by cell cultures of *C. argentea* upon methyl jasmonate elicitation at various concentrations, and the results are illustrated in Fig. 5.

A pronounced increase in betalains content was observed in suspension cultures treated with 50 and 100 µM methyl jasmonate, yielding the maximum values of 3.32 and 3.23 mg/g DW, respectively. Increasing the concentrations of methyl jasmonate to 150 and 200 µM resulted in a decline of betalains content compared with the control treatment without elicitation (Fig. 5A). These findings are in accordance with previous studies. Bhuiyan and Adachi^54^ demonstrated that exogenous application of methyl jasmonate significantly increased betacyanin synthesis in cell suspension cultures of *Portulaca* sp. cv. Jewel. Suresh et al.^55^ also noted that methyl jasmonate enhanced the production of betalains in hairy root cultures of *B. vulgaris*. Furthermore, Lakhotia et al.^13^ pointed out that methyl jasmonate at a low concentration of 0.5 µM significantly enhanced the production of betacyanin and betaxanthin in callus cultures of *Bougainvillea* spp.

It is evident from the literature that methyl jasmonate induces the accumulation of plant secondary metabolites by activating the expression of several genes involved in defense response mechanisms as well as those genes involved in the biosynthesis pathway of each bioactive compound ^56,57^. Based on this information, the increase in betalains content in cell suspension cultures of *C. argentea* after elicitation with methyl jasmonate might be associated with the expression of the genes encoding enzymes involved in the betalains biosynthesis pathway. To clarify this matter, further study on expression analysis of genes involved in the betalains biosynthesis pathway should be performed in the cell cultures of *C. argentea* after methyl jasmonate elicitation.

The betalains content and antioxidant activity detected in the methyl jasmonate elicited cells are closely correlated. High betalains content, especially from suspension cultures treated with 50 and 100 µM methyl jasmonate, displayed slightly higher DPPH and ABTS activities than the control and other treated cells (Fig. 5B). This finding indicated that the enhancement of betalains production after methyl jasmonate elicitation led to an increase in the antioxidant property, similar to other studies previously reported ^22,23,58^.

In addition to phytohormones, several microelements, such as cadmium chloride (CdCl₂), silver nitrate (AgNO₃), calcium chloride (CaCl₂), manganese sulfate (MnSO₄), zinc sulfate (ZnSO₄), copper sulfate (CuSO₄), and cobalt chloride (CoCl₂), have also been used as abiotic elicitors to improve the synthesis of phytochemicals ^13−15,19,24^. Although different plant species or types of culture systems respond to each microelement differently, CuSO₄ and CoCl₂ exhibited promising effects in enhancing the production of several plant secondary metabolites. Therefore, in this study, CuSO₄ and CoCl₂ were chosen, and their biological impact on betalains production in cell suspension cultures of *C. argentea* was determined.

**Fig. 6:**
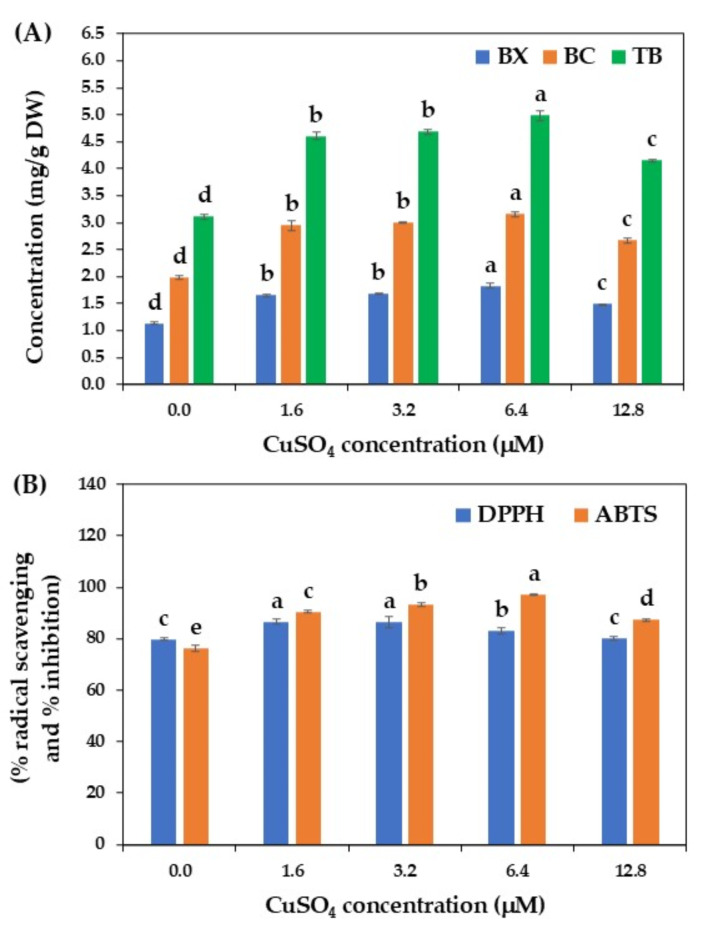
Effect of CuSO_4_ on betalains production by cell suspension cultures of *C. argentea*. (A) betalains production and (B) antioxidant activity. BX is betaxanthin, BC is betacyanin, and TB is total betalains. Bars represent means ± standard deviations (SDs) from two independent experiments, each with five replications. For each parameter, different letters above the bars indicate statistically significant differences between treatments (*p* < 0.05).

As shown in Fig. 6A, the addition of CuSO₄ into the culture medium significantly increased betalains production. The highest betalains content (4.99 mg/g DW) was detected in suspension cultures treated with 6.4 µM CuSO₄, which was approximately 1.6-fold higher than the control treatment without elicitation. The enhancement of betalains production in suspension cultures of *C. argentea* may be attributed to the increase in activity of enzymes involved in the biosynthesis pathway of betalains since CuSO₄ can act as a cofactor for many enzymes, as previously proposed by Lakhotia et al.^13^ and Zarad et al.^59^. The present results are in harmony with a study by Lakhotia et al.^13^, who reported that the addition of CuSO₄, specifically at 20 µM, significantly increased the accumulation of betacyanin and betaxanthin in callus cultures of *Bougainvillea* spp. Trejo-Tapia et al. ^12^ also pointed out that CuSO₄ at 5 µM could enhance betalains production in suspension cultures of *B. vulgaris*. Notably, the opposite result was also recorded by Warhade and Badere^14^, where the addition of CuSO₄ dramatically reduced the synthesis of amaranthin, betanin, batalamic acid, and betaxanthin in cell suspension cultures of *C. cristata*.

It should be noted in the current study that the betalains content tended to decline when suspension cultures of *C. argentea* were treated with a high concentration of CuSO₄ (12.8 µM). One possibility is that a high concentration of CuSO₄ may be toxic to plant cells and can inhibit the activity of some enzymes involved in the biosynthesis pathway of betalains ^14,59^.

Strong DPPH and ABTS antioxidant activities were detected in the betalains samples after CuSO₄ elicitation, as compared to the control. Overall, the highest DPPH activity (86.62% radical scavenging) was recorded in cell cultures treated with 1.6 µM CuSO₄, while the highest ABTS activity (97.13% inhibition) was detected in the cell cultures treated with 6.4 µM CuSO₄. The control sample without elicitation yielded the highest DPPH and ABTS activities of 79.78% radical scavenging and 76.29% inhibition, respectively (Fig. 6B). It can be concluded based on these findings that CuSO₄ enhanced betalains accumulation in cell suspension cultures of *C. argentea* and consequently increased the DPPH and ABTS antioxidant properties of these compounds, in line with the results reported by Lala ^60^ and Zarad et al.^59^.

Considering CoCl_2_, this microelement plays a crucial role in various plant physiological processes, such as cell growth and development, respiration, and nucleic acid synthesis^61^. It is also an essential factor in many enzymes and coenzymes responsible for the production of plant secondary metabolites^59^. CoCl_2_ has been widely used as an abiotic elicitor to trigger the production of several phytoconstituents, such as betalains in suspension cultures of *B. vulgaris*^12^ and *C. cristata*^14^, paclitaxel in suspension cultures of *Taxus* spp.^62^, hesperidin and robinin in callus cultures of *Cordia myxa*^63^, and phenolic compounds in callus cultures of *Artemisia annua*^59^. Therefore, this study investigated the effect of CoCl_2_ elicitation on betalains production in cell suspension cultures of *C. argentea*.

**Fig. 7:**
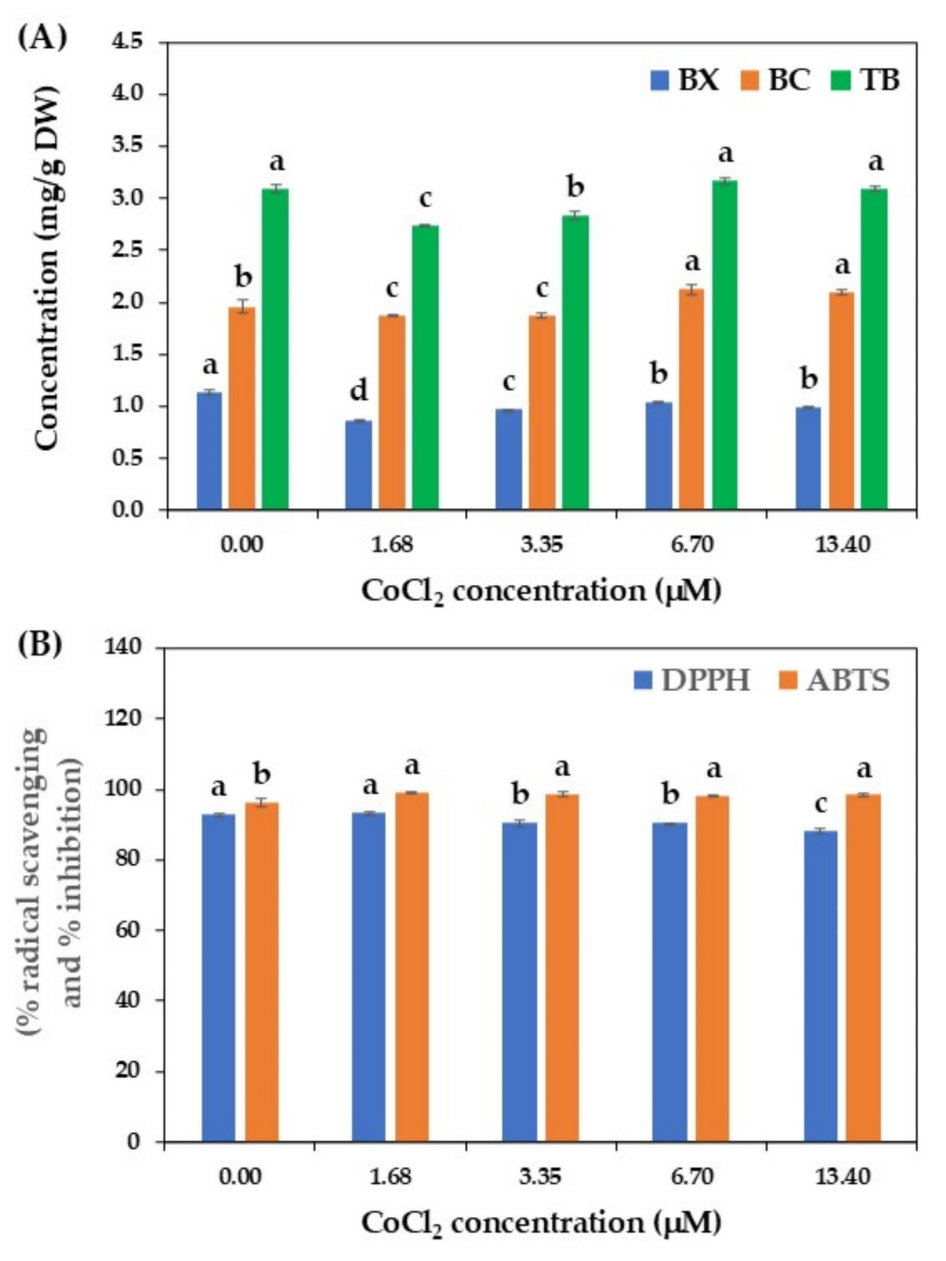
Effect of CoCl_2_ on betalains production by cell suspension cultures of *C. argentea*. (A) betalains production and (B) antioxidant activity. BX is betaxanthin, BC is betacyanin, and TB is total betalains. Bars represent means ± standard deviations (SDs) from two independent experiments, each with five replications. For each parameter, different letters above the bars indicate statistically significant differences between treatments (*p* < 0.05).

Based on a study by Warhade and Badere^14^, various concentrations of CoCl_2_ were applied in this study. Insignificant differences in betalains accumulation were recorded in cell cultures treated with CoCl_2_ compared to the control (Fig. 7A). The betalains content from CoCl_2_-treated cells ranged from 2.74 to 3.16 mg/g DW, while that from the control cells was 3.09 mg/g DW. These findings differ from those reported for the production of betalains in *B. vulgaris* ^12^, amaranthin, betanin, betalamic acid, and betaxanthin in *C. cristata*^14^, and phenolic compounds in *A. annua*^59^, where CoCl_2_ enhanced the production of those bioactive compounds.

The difference in response to CoCl_2_ might be due to variations in plant species, the stage of plant growth for applying elicitors, and the type of culture systems. For instance, Warhade and Badere^14^ reported that adding 6.7 µM CoCl_2_ to the suspension cultures of *C. cristata* after 12 days of cultivation significantly enhanced the accumulation of amaranthin, betanin, betalamic acid, and betaxanthin. Trejo-Tapia et al.^12^ found that adding 5 µM CoCl_2_ at the beginning of cultivation (day 0) improved betalains production in suspension cultures of *B. vulgaris*. The recent study by Zarad et al.^59^ also pointed out that elicitation of *A. annua* callus cultures with 2 mg/L CoCl_2_ yielded the highest phenolic content, approximately 126% greater than that of the control cells.

Like betalain content, CoCl_2_ elicitation did not enhance antioxidant activity in treated culture cells compared to the control. Betalains from CoCl₂-elicited cultures exhibited DPPH radical scavenging (88.30 − 93.30%) and ABTS inhibition (98.15 − 99.27%) activities similar to those of the control treatment (Fig. 7B). These findings suggest that CoCl_2_ had no significant effect on betalain production and antioxidant properties in *C. argentea* cell suspension cultures, contrasting with previous reports on callus cultures ⁵⁹. This discrepancy in CoCl_2_ response may be attributed to differences in plant species and cell culture systems.

The study on *Celosia argentea* cell suspension cultures demonstrates that both biotic and abiotic elicitors significantly enhance betalains production compared to the control (Table 1). Among the tested elicitors, CuSO₄ (an abiotic elicitor) proved most effective, increasing total betalains by 100.2%, followed closely by chitosan (a biotic elicitor) with an 86.9% increase. This finding suggests that the specific nature of the elicitor, rather than its broad classification as biotic or abiotic, is crucial for its effectiveness. The effectiveness ranking of elicitors, from highest to lowest, is as follows: CuSO₄, chitosan, salicylic acid, methyl jasmonate, CoCl₂, and yeast extract. Notably, betacyanin levels consistently exceeded betaxanthin levels across all treatments, maintaining a relatively stable ratio.

The superior performance of CuSO₄ and chitosan may be attributed to their roles in stress signaling pathways. CuSO₄ potentially triggers antioxidant responses, while chitosan might mimic pathogen attacks, thereby activating plant defense mechanisms. These insights have practical implications for industrial betalains production, with CuSO₄ treatment appearing most promising, while chitosan offers an effective organic alternative.

**Table 1:** Comparative analysis of biotic and abiotic elicitors on betalains production in cell suspension cultures of *C. argentea*.

Elicitors	Betaxanthin (mg/g DW)	Betacyanin (mg/g DW)	Total betalains (mg/g DW)
Control without elicitors	0.971 ± 0.013 ^e^	1.519 ± 0.011 ^f^	2.490 ± 0.022 ^f^
*Biotic elicitors*			
Chitosan	1.757 ± 0.050 ^b^	2.897 ± 0.047 ^b^	4.654 ± 0.085 ^b^
Yeast extract	1.013 ± 0.053 ^e^	2.005 ± 0.079 ^de^	3.017 ± 0.126 ^e^
*Abiotic elicitors*			
Salicylic acid	1.400 ± 0.029 ^c^	2.431 ± 0.019 ^c^	3.863 ± 0.045 ^c^
Methyl jasmonate	1.330 ± 0.002 ^d^	1.990 ± 0.008 ^e^	3.320 ± 0.013 ^d^
CuSO_4_	1.831 ± 0.045 ^a^	3.155 ± 0.053 ^a^	4.986 ± 0.090 ^a^
CoCl_2_	1.037 ± 0.010 ^e^	2.217 ± 0.048 ^d^	3.164 ± 0.031 ^de^

Values represent means ± standard deviations (SDs) from two independent experiments, each conducted with five replicates. Different letters following the values indicate statistically significant differences between treatments (*p* < 0.05).

This study demonstrates that elicitation using appropriate concentrations of biotic or abiotic elicitors can improve both the production of betalains and their antioxidant activity in *C. argentea* cell suspension cultures. However, the research also highlights several potential directions for future investigations, including examining the time course of betalain production after elicitor treatment and exploring synergistic effects of combined elicitors.

While this study provides valuable insights, it is important to note that other techniques could also induce the production and accumulation of plant secondary metabolites. Based on existing literature, these techniques include the application of light and the addition of microbial polysaccharides, amino acids, or precursors for secondary metabolite production. Therefore, further research exploring these alternative methods could yield valuable insights into optimizing betalain production in plant cell cultures.

Since the biosynthesis pathway of betalains in plant cells is complex and involves several genes and enzymes, and the exact regulatory mechanisms of plant cells in response to biotic and abiotic elicitors related to betalains production are also limited, further investigation should be carried out to explore the expression of genes or the enzyme activity involved in defense mechanisms or biosynthesis pathways of betalains in *C. argentea* cell cultures after elicitation. This information will be useful in designing an appropriate and effective strategy to improve betalains production for this type of cell culture system in this plant.

## Conclusion

Elicitation is one of the most promising approaches for enhancing the production of plant secondary metabolites. As demonstrated in the current study, biotic and abiotic elicitors could increase the production and accumulation of betalains in cell suspension cultures of *C. argentea*. Chitosan at 5.0 mg/L and CuSO_4_ at 6.4 µM were proven to be the most effective biotic and abiotic elicitors in enhancing the in vitro production of betalains, yielding the maximum betalains content of 4.65 and 4.99 mg/g, respectively. In addition, the antioxidant activity of betalains extracted from culture cells treated with these elicitors also significantly increased. The results established in this study, which have never been reported before, can be applied for sustainable in vitro production of betalains from *C. argentea* on a commercial scale, thereby expanding its application in various industries, such as foods and beverages, textiles, cosmetics, and pharmaceuticals.

### Submission declaration and verification

Submission of an article implies that the work described has not been published previously in any form.

## Data Availability

All data generated or analyzed during this study are included in this published article.

## References

[CR1] 1. Carreón-Hidalgo, J.P., Franco-Vásquez, D.C., Gómez-Linton, D.R. & Pérez-Flores, L.J. Batalain plant sources, biosynthesis, extraction, stability enhancement methods, bioactivity, and applications. *Food Res. Int*. **151**, 110821 (2022). https://doi.org/10.1016/j.foodres.2021.110821. 10.1016/j.foodres.2021.110821 34980373

[CR2] 2. Khan, M.I. & Giridhar, P. Plant betalains: chemistry and biochemistry. *Phytochem*. **117**, 267−295 (2015). https://doi.org/10.1016/j.phytochem.2015.06.008. 10.1016/j.phytochem.2015.06.008 26101148

[CR3] 3. Khan, M.I. Stabilization of betalains: a review. *Food Chem*. **197**, 1280−1285 (2016). https://doi.org/10.1016/j.foodchem.2015.11.013. 10.1016/j.foodchem.2015.11.043 26675869

[CR4] 4. Rodriguez-Amaya, D.B. Natural food pigments and colorants. In *Bioactive molecules in food, Reference series in phytochemistry* (eds Mérillon, J.M. & Ramawat, K.G.) 1−35 (Springer International Publishing, 2018). https://doi.org/10.1016/j.foodres.2018.05.028.

[CR5] 5. Pavokovic, D. & Krsnik-Rasol, M. Complex biochemistry and biotechnological production of betalains. *Food Technol. Biotechnol*. **49**, 145–155 (2011).

[CR6] 6. Thorat, B.R. Review on *Celosia argentea* L. plant. *Res. J. Pharmacognosy Phytochem*. **10**, 109−119 (2018). https://doi.org/10.5958/0975-4385.2018.00017.1.

[CR7] 7. Divya, B.J., Jyothi Sravani, M., Hari Chandana, J., Sumana, T. & Thyagaraju, K. Phytochemical and phytotherapeutic activities of *Celosia argentea*: a review. *World J. Pharm. Pharmaceu. Sci*. **8**, 488−505 (2019). https://doi.org/10.20959/wjpps20193-13312.

[CR8] 8. Schliemann, W., Cai, Y., Degenkolb, T., Schmidt, J. & Corke, H. Betalains of *Celosia argentea*. *Phytochemistry*. **58**, 159–165 (2001). https://doi.org/10.1016/s0031-9422(01)00141-8. 10.1016/s0031-9422(01)00141-8 11524126

[CR9] 9. Leathers, R.R., Davin, C. & Zrÿd, J.P. Betalain producing cell cultures of *Beta vulgaris* L. var. Bikores monogerm (red beet). *In Vitro Cell Dev. Biol.-Plant*. **28**, 39−45 (1992). https://doi.org/10.1007/BF02823016.

[CR10] 10. Rodriguez, M., Jimenez, A.A., Davila, O.G. & Sepulveda, J.G. Effect of carbon source on cell growth and betalains production in cell suspension culture of *Beta vulgaris*. *Biotechnol. Lett*. **16**, 853−858 (1994). https://doi.org/10.1007/BF00133967.

[CR11] 11. Trejo-Tapia, G., Jimenez-Aparicio, A., Rodriguez, M., Sepulveda, G., Salcedo, G., Martinez, B., Gutierrez, G. & De Jesus, A. Influence of medium constituents on growth and betalain production in cell suspension cultures of *Beta vulgaris*. *Asia-Pacific J. Mol. Biol. Biotechnol*. **7**, 167−172 (1999).

[CR12] 12. Trejo-Tapia, G., Jimenez-Aparicio, A., Rodriguez-Monroy, M., De Jesus-Sanchez, A. & Gutierrez-Lopez, G. Influence of cobalt and other microelements on the production of betalains and the growth of suspension cultures of *Beta vulgaris*. *Plant Cell Tiss. Organ Cult*. **67**, 19−23 (2001). https://doi.org/10.1023/A:1011684619614.

[CR13] 13. Lakhotia, P., Singh, K.P., Singh, S.K., Singh, M.C., Prasad, K.V. & Swaroop, K. Influence of biotic and abiotic elicitors on production of betalain pigments in bougainvillea callus cultures. *Indian J. Hort*. **71**, 373−378 (2014).

[CR14] 14. Warhade, M.I. & Badere R.S. *Fusarium oxysporum* cell elicitor enhances betalain content in the cell suspension culture of *Celosia cristata*. *Physiol. Mol. Biol. Plants*. **24**, 285−293 (2018). https://doi:10.1007/s12298-018-0511-x. 10.1007/s12298-018-0511-x PMC5834993 29515322

[CR15] 15. Winson, K.W.S., Chew, B.L., Sathasivam, K. & Subramaniam, S. Effect of amino acid supplementation, elicitation and LEDs on *Hylocereus costaricensis* callus culture for the enhancement of betalain pigments. *Scientia Horticulturae*. **289**, 110459 (2021). https://doi.org/10.1016/j.scienta.2021.110459.

[CR16] 16. Sharma, M., Ahuja, A., Gupta, R. & Mallubhotla, S. Enhanced bacoside production in shoot cultures of *Bacopa monnieri* under the influence of abiotic elicitors. *Nat. Prod. Res*. **29**, 745−749 (2015). https://doi.org/10.1080/14786419.2014.986657. 10.1080/14786419.2014.986657 25485652

[CR17] 17. Ramirez-Estrada, K., Vidal-Limon, H., Hidalgo, D., Moyano, E., Golenioswki, M., Cusidó, R.M. & Palazon, J. Elicitation, an effective strategy for the biotechnological production of bioactive high-added value compounds in plant cell factories. *Molecules.* **21**, 182 (2016). https://doi.org/10.3390/molecules21020182. 10.3390/molecules21020182 PMC6273650 26848649

[CR18] 18. Fooladi vanda, G., Shabani, L. & Razavizadeh, R. Chitosan enhances rosmarinic acid production in shoot cultures of *Melissa officinalis* L. through the induction of methyl jasmonate. *Bot. Stud*. **60**, 26 (2019). https://doi.org/10.1186/s40529-019-0274-x. 10.1186/s40529-019-0274-x PMC6797681 31624938

[CR19] 19. Al-Khayri, J.M. & Naik, P.M. Elicitor-induced production of biomass and pharmaceutical phenolic compounds in cell suspension culture of date palm (*Phoenix dactylifera* L.). *Molecules*. **25**, 4669 (2020). https://doi.org/10.3390/molecules25204669. 10.3390/molecules25204669 PMC7587379 33066253

[CR20] 20. El-Ashry, A.A.E., El-Bahr, M.K. & Gabr, A.M.M. Effect of light quality on betalain content of red beet (*Beta vulgaris* L.) cultured *in vitro*. *Egyptian Pharm. J*. **19**, 143–148 (2020). https://doi.org/10.4103/epj.epj_43_19.

[CR21] 21. Shah, M., Jan, H., Drouet, S., Tungmunnithum, D., Shirazi, J.H., Hano, C. & Abbasi, B.H. Chitosan elicitation impacts flavonolignan biosynthesis in *Silybum marianum* (L.) Gaertn cell suspension and enhances antioxidant and anti-inflammatory activities of cell extracts. *Molecules*. **26**, 791 (2021). https://doi.org/10.3390/molecules26040791. 10.3390/molecules26040791 PMC7913645 33546424

[CR22] 22. Mendoza, D., Causpud, O., Arias, J.P., Ruiz, O. & Arias, M. Effect of salicylic acid and methyl jasmonate in the production of phenolic compounds in plant cell suspension cultures of *Thevetia peruviana*. *Biotechnol. Rep*. **19**, e00273 (2018). https://doi.org/10.1016/j.btre.2018.e00273. 10.1016/j.btre.2018.e00273 PMC6039307 29998072

[CR23] 23. Autaijamsripon, J., Jirakiattikul, Y., Rithichai, P. & Itharat, A. Effect of phenylalanine and methyl jasmonate on secondary metabolite production by shoot cultures of holy basil, purple-type (*Ocimum sanctum* L.). *Sci. Technol. Asia*. **28**, 229−239 (2023). https://doi.org/10.14456/scitechasia.2023.19.

[CR24] 24. Jeyasri, R., Muthuramalingam, P., Karthick, K., Shin, H., Choi, S.H. & Ramesh, M. Methyl jasmonate and salicylic acid as powerful elicitors for enhancing the production of secondary metabolites in medicinal plants: an updated review. *Plant Cell Tiss. Organ Cult*. **153**, 447−458 (2023). https://doi.org/10.1007/s11240-023-02485-8. 10.1007/s11240-023-02485-8 PMC10026785 37197003

[CR25] 25. Gorni, P.H., Gonçalves, L.S., Spera, K.D., Pacheco, A.C. & Lapaz, A.M. Exogenous salicylic acid increases productivity and elicits betalains and other bioactive compounds in red beetroot. *Trop. Plant Biol*. **16**, 41−52 (2023). https://doi.org/10.1007/s12042-023-09329-x.

[CR26] 26. Henarejos-Escudero, P., Guadarrama-Flores, B., Guerrero-Rubio, M.A., Gómez-Pando, L.R., García-Carmona, F. & Gandía-Herrero, F. Development of betalain producing callus lines from colored quinoa varieties (*Chenopodium quinoa* Willd). *J. Agric. Food Chem*. **66**, 467–474 (2018). https://doi.org/10.1021/acs.jafc.7b04642. 10.1021/acs.jafc.7b04642 29239176

[CR27] 27. Visockis, M., Bobinaite, R., Ruzgys, P., Barakauskas, J., Markevičius, V., Viškelis, P. & Šatkauskas, S. Assessment of plant tissue disintegration degree and its related implications in the pulsed electric field (PEF)-assisted aqueous extraction of betalains from the fresh red beetroot. *Innov. Food Sci. Emerg*. **73**, 102761 (2021). https://doi.org/10.1016/j.ifset.2021.102761.

[CR28] 28. Chandran, H., Meena, M., Barupal, T. & Sharma, K. Plant tissue culture as a perpetual source for production of industrially important bioactive compounds. *Biotechnol. Rep*. **26**, e00450 (2020). https://doi.org/10.1016/j.btre.2020.e00450. 10.1016/j.btre.2020.e00450 PMC7193120 32373483

[CR29] 29. Choi, J.W., Kim, Y.K., Lee, W.H., Pedersen, H. & Chin, C.K. Kinetic model of cell growth and secondary metabolite synthesis in plant cell culture of *Thalictrum rugosum*. *Biotechnol. Bioprocess Eng*. **4**, 129−137 (1999). https://doi.org/10.1007/BF02932383.

[CR30] 30. Siatka, T. Production of anthocyanins in callus cultures of *Angelica archangelica*. *Nat. Prod. Commun*. **13**, 1645−1648 (2018). https://doi.org/10.1177/1934578X1801301219.

[CR31] 31. Linh, T.M., Mai, N.C., Hoe, P.T., Ngoc, N.T., Thao, P.T.H., Ban, N.K. & Van, N.T. Development of a cell suspension culture system for promoting alkaloid and vinca alkaloid biosynthesis using endophytic fungi isolated from local *Catharanthus roseus*. *Plants.* **10**, 672 (2021). https://doi.org/10.3390/plants10040672. 10.3390/plants10040672 PMC8066771 33807415

[CR32] 32. Cusido, R.M., Palazon, J., Bonfill, M., Navia-Osorio, A., Morales, C. & Piñol, M.T. Improved paclitaxel and baccatin III production in suspension cultures of *Taxus media*. *Biotechnol. Prog*. **18**, 418−423 (2002). https://doi.org/10.1021/bp0101583. 10.1021/bp0101583 12052053

[CR33] 33. Malik, S., Cusido, R.M., Mirjalili, M.H., Moyano, E., Palazon, J. & Bonfill, M. Production of the anticancer drug taxol in *Taxus baccata* suspension cultures: A review. *Process Biochem*. **46**, 23−34 (2011). https://doi.org/10.1016/j.procbio.2010.09.004.

[CR34] 34. Ferri, M. & Tassoni, A. Chitosan as elicitor of health beneficial secondary metabolites in *in vitro* plant cell cultures. In *Handbook of chitosan research and applications* (eds Mackay, R.G. & Tait, J.M.) 389−413 (Nova Science Publishers, Inc., 2011).

[CR35] 35. Komaraiah, P., Naga Amrutha, R.N., Kavi Kishor, P.B. & Ramakrishna, S.V. Elicitor enhanced production of plumbagin in suspension cultures of *Plumbago rosea* L. *Enz. Microb. Technol*. **31**, 634−639 (2002). https://doi.org/10.1016/S0141-0229(02)00159-X.

[CR36] 36. Ferri, M., Tassoni, A., Franceschetti, M., Righetti, L., Naldrett, M.J. & Bagni, N. Chitosan treatment induces changes of protein expression profile and stilbene distribution in *Vitis vinifera* cell suspensions. *Proteomics*. **9**, 610−624 (2009). https://doi.org/10.1002/pmic.200800386. 10.1002/pmic.200800386 19132683

[CR37] 37. Gabr, A.M.M., Mabrok, H.B., Ghanem, K.Z., Blaut, M. & Smetanska, I. Lignan accumulation in callus and *Agrobacterium rhizogenes*-mediated hairy root cultures of flax (*Linum usitatissimum*). *Plant Cell Tiss. Organ Cult*. **126**, 255−267 (2016). https://doi.org/10.1007/s11240-016-0995-4.

[CR38] 38. Kahromi, S. & Khara, J. Chitosan stimulates secondary metabolite production and nutrient uptake in medicinal plant *Dracocephalum kotschyi*. *J. Sci. Food Agric*. **101**, 3898−3907 (2021). https://doi.org/10.1002/jsfa.11030. 10.1002/jsfa.11030 33348431

[CR39] 39. Tao, Z., Yuan, H., Liu, M., Liu, Q., Zhang, S., Liu, H., Jiang, Y., Huang, D. & Wang, T. Yeast extract: characteristics, production, applications and future perspectives. *J. Microbiol. Biotechnol*. **33**, 151−166 (2023). https://doi.org/10.4014/jmb.2207.07057. 10.4014/jmb.2207.07057 PMC9998214 36474327

[CR40] 40. Shams-Ardakani, M., Hemmati, S. & Mohagheghzadeh, A. Effect of elicitors on the enhancement of podophyllotoxin biosynthesis in suspension cultures of *Linum album*. *DARU J. Pharm. Sci*. **13**, 56−60 (2005).

[CR41] 41. Sánchez-Sampedro, M.A., Fernández-Tárrago, J. & Corchete, P. Yeast extract and methyl jasmonate-induced silymarin production in cell cultures of *Silybum marianum* (L.) Gaertn. *J. Biotechnol*. **119**, 60−69 (2005). https://doi.org/10.1016/j.jbiotec.2005.06.012. 10.1016/j.jbiotec.2005.06.012 16054261

[CR42] 42. Roat, C. & Ramawat, K.G. Elicitor-induced accumulation of stilbenes in cell suspension cultures of *Cayratia trifolia* (L.) Domin. *Plant Biotechnol. Rep*. **3**, 135−138 (2009). https://doi.org/10.1007/s11816-009-0082-y.

[CR43] 43. Ali, B. Salicylic acid: an efficient elicitor of secondary metabolite production in plants. *Biocatal. Agric. Biotechnol*. **31**, 101884 (2021). https://doi.org/10.1016/j.bcab.2020.101884.

[CR44] 44. Namdeo, A.G. Plant cell elicitation for production of secondary metabolites: A review. *Pharmacogn. Rev*. **1**, 69−79 (2007).

[CR45] 45. Xu, A., Zhan, J.C. & Huang, W.D. Effects of ultraviolet C, methyl jasmonate and salicylic acid, alone or in combination, on stilbene biosynthesis in cell suspension cultures of *Vitis vinifera* L. cv. Cabernet Sauvignon. *Plant Cell Tiss. Organ Cult*. **122**, 197−211 (2015). https://doi.org/10.1007/s11240-015-0761-z.

[CR46] 46. Acikgoz, M.A., Kara, S.M., Aygun, A.H.M.E.T., Ozcan, M.M. & Ay, E.B. Effect of methyl jasmonate and salicylic acid on the production of camphor and phenolic compounds in cell suspension culture of endemic Turkish yarrow (*Achillea gypsicola*) species. *Turkish J. Agric. Forestry*. **43**, 351−359 (2019). https://doi.org/10.3906/tar-1809-54.

[CR47] 47. Koul, A. & Mallubhotla, S. Elicitation and enhancement of bacoside production using suspension cultures of *Bacopa monnieri* (L.) Wettst. *3 Biotech*. **10**, 1−4 (2020). https://doi.org/10.1007/s13205-020-02242-0. 10.1007/s13205-020-02242-0 PMC7230074 32432018

[CR48] 48. Rajan, M., Feba, K.S., Chandran, V., Shahena, S. & Mathew, L. Enhancement of rhamnetin production in *Vernonia anthelmintica* (L.) Willd. cell suspension cultures by eliciting with methyl jasmonate and salicylic acid. *Physiol. Mol. Biol. Plants*. **26**, 1531−1539 (2020). https://doi.org/10.1007/s12298-020-00829-8. 10.1007/s12298-020-00829-8 PMC7326840 32647466

[CR49] 49. Kandoudi, W. & Németh-Zámboriné, E. Stimulating secondary compound accumulation by elicitation: Is it a realistic tool in medicinal plants in vivo? *Phytochem. Rev*. **1**, 19 (2022). https://doi.org/10.1007/s11101-022-09822-3.

[CR50] 50. Ho, T.T., Murthy, H.N. & Park, S.Y. Methyl jasmonate induced oxidative stress and accumulation of secondary metabolites in plant cell and organ cultures. *Int. J. Mol. Sci*. **21**, 716 (2020). https://doi.org/10.3390/ijms21030716. 10.3390/ijms21030716 PMC7037436 31979071

[CR51] 51. Wang, Y., Mostafa, S., Zeng, W. & Jin, B. Function and mechanism of jasmonic acid in plant responses to abiotic and biotic stresses. *Int. J. Mol. Sci*. **22**, 8568 (2021). https://doi.org/10.3390/ijms22168568. 10.3390/ijms22168568 PMC8395333 34445272

[CR52] 52. Chodisetti, B., Rao, K., Gandi, S. & Giri, A. Gymnemic acid enhancement in the suspension cultures of *Gymnema sylvestre* by using the signaling molecules-methyl jasmonate and salicylic acid. *In Vitro Cell. Dev. Biol.-Plant*. **51**, 88−92 (2015). https://doi.org/10.1007/s11627-014-9655-8.

[CR53] 53. Wang, J., Qian, J., Yao, L. & Lu, Y. Enhanced production of flavonoids by methyl jasmonate elicitation in cell suspension culture of *Hypericum perforatum*. *Bioresour. Bioprocess*. **2**, 1−9 (2015). https://doi.org/10.1186/s40643-014-0033-5.

[CR54] 54. Bhuiyan, N.H. & Adachi, T. Stimulation of betacyanin synthesis through exogenous methyl jasmonate and other elicitors in suspension-cultured cells of *Portulaca*. *J. Plant Physiol*. **160**, 1117−1124 (2003). https://doi.org/10.1078/0176-1617-01044. 10.1078/0176-1617-01044 14593814

[CR55] 55. Suresh, B., Thimmaraju, R., Bhagyalakshmi, N. & Ravishankar, G.A. Polyamine and methyl jasmonate-induced enhancement of betalain production in hairy root cultures of *Beta vulgaris* grown in bubble column reactor and studies on efflux of pigments. *Proc. Biochem*. **39**, 2091−2096 (2004). https://doi.org/10.1016/j.procbio.2003.10.009.

[CR56] 56. Chaichana, N. & Dheeranupattana, S. Effects of methyl jasmonate and salicylic acid on alkaloid production from *in vitro* culture of *Stemona* sp. *Int. J. Biosci. Biochem. Bioinform*. **2**, 146−150 (2012). https://doi.org/10.7763/IJBBB.2012.V2.89.

[CR57] 57. Rubio-Rodriguez, E., Vera-Reyes, I., Sepúlveda-Garcia, E.B., Ramos-Valdivia, A.C. & Trejo-Tapia, G. Secondary metabolite production and related biosynthetic genes expression in response to methyl jasmonate in *Castilleja tenuiflora* Benth. *in vitro* plants. *Plant Cell Tiss. Organ Cult*. **144**, 519−532 (2021). https://doi.org/10.1007/s11240-020-01975-3.

[CR58] 58. Danaee, M., Farzinebrahimi, R., Kadir, M.A., Sinniah, U.R., Mohamad, R. & Taha, R.M. Effects of MeJA and SA elicitation on secondary metabolic activity, antioxidant content and callogenesis in *Phyllanthus pulcher*. *Braz. J. Bot*. **38**, 265−272 (2015). https://doi.org/10.1007/s40415-015-0140-3.

[CR59] 59. Zarad, M.M., Toaima, N.M., Refaey, K.A., Atta, R.F. & Elateeq, A.A. Copper sulfate and cobalt chloride effect on total phenolics accumulation and antioxidant activity of *Artemisia annua* L. callus cultures. *Al-Azhar J. Agri. Res*. **46**, 26−40 (2021). https://10.21608/AJAR.2021.245610.

[CR60] 60. Lala, S. Enhancement of secondary metabolites in *Bacopa monnieri* (L.) Pennell plants treated with copper-based nanoparticles *in vivo*. *IET Nanobiotechnol*. **14**, 78−85 (2020). https://doi.org/10.1049/iet-nbt.2019.0124. 10.1049/iet-nbt.2019.0124 PMC8675962 31935682

[CR61] 61. Amarasinghe, A.A.Y. Effects of copper sulphate and cobalt chloride on in vitro performances of traditional indica rice (*Oryza sativa* L.) varieties in Sri Lanka. *J. Agric. Sci*. **4**, 132−141 (2009). https://doi.org/10.4038/jas.v4i3.1652.

[CR62] 62. Zhang, C.H. & Wu, J.Y. Ethylene inhibitors enhance elicitor-induced paclitaxel production in suspension cultures of *Taxus* spp. cells. *Enzyme Microb. Technol*. **32**, 71−77 (2003). https://doi.org/10.1016/S0141-0229(02)00266-1.

[CR63] 63. Taha, A.J. Effect of some chemical elicitors on some secondary metabolite induction of *Cordia myxa* L. *in vitro*. *IOSR J. Pharm*. **6**, 15−20 (2016).

